# LncRNA 2310043L19Rik inhibits differentiation and promotes proliferation of myoblast by sponging miR-125a-5p

**DOI:** 10.18632/aging.102905

**Published:** 2020-03-31

**Authors:** Rongyang Li, Bojiang Li, Ming Shen, Yan Cao, Xuan Zhang, Weijian Li, Jingli Tao, Wangjun Wu, Honglin Liu

**Affiliations:** 1 Department of Animal Genetics, Breeding and Reproduction, College of Animal Science and Technology, Nanjing Agricultural University, Nanjing 210095, China; 2 College of Animal Science and Veterinary Medicine, Shenyang Agricultural University, Shenyang 110866, China

**Keywords:** lncRNA, ceRNA, miR-125a-5p, E2F3, myoblast

## Abstract

Although many long non-coding RNAs (lncRNAs) have been identified in muscle, some of their physiological functions and regulatory mechanisms remain elusive. Here we report the functional identification and characterization of a novel lncRNA 2310043L19Rik (lnc-231), which is highly expressed in muscle. The expression level of lnc-231 in skeletal muscle of young mice is higher than that in aged mice. Functional analysis showed that overexpression of lnc-231 restrained differentiation and promoted proliferation of myoblast, while inhibition of lnc-231 revealed completely opposite effects *in vitro*. RNA molecules of lnc-231 acted mechanistically as competing endogenous RNAs (ceRNA) to target miR-125a-5p, whereas miR-125a-5p binds to the 3’-UTR of E2F3 mRNA to inhibit its function. Collectively, lncRNA 2310043L19Rik promotes proliferation and inhibits differentiation of myoblast cells by attenuating the function of miR-125a-5p.

## INTRODUCTION

Long non-coding RNAs are a class of RNAs longer than 200 nt with lower protein coding potential. They are located in the intergenic region that does not overlap with the annotated coding genes. LncRNA was originally proposed in the study of universal transcription of unknown coding regions by chip [1]. In the early 1990s, LincRNA Xist was found to be inactivated on the X chromosome (XCI) [2–4]. and it can inhibit the transcription of HOTAIR HOX family gene [5].

Skeletal muscle, the most plentiful tissue in domestic animals, is required for maintaining body movement and provides meat products to humans. The different regulatory mechanisms during the growth and development of skeletal muscle cause differences in muscle yield and meat quality. The growth and development of skeletal muscle is a complex process involving the proliferation, migration, differentiation, and the fusion of muscle stem cells, as well as the formation of muscle fibers [6]. Adult muscles have a strong adaptation capacity, enabling functional switches in response to altered conditions. For instance, adult skeletal muscles will change and adjust their functions during aging [7]. Deletion of small ankyrin 1 (sAnk1) isoforms results in structural and functional alterations in aging skeletal muscle fibers [8]. Deletion of pofut1 in mouse skeletal myofibers induces muscle aging-related phenotypes [9]. Moreover, there is a close correlationship between lncRNA expression and aging [10].

Studies of lncRNA functions have been carried out extensively in specific cell types, developmental stages, and diseases [11]. It has been demonstrated that lincRNAs play important roles in skeletal muscle growth and development, muscle cell proliferation, migration, differentiation, and apoptosis [12].

Myogenesis is also regulated by some functional lncRNAs, including SRA, Gtl2 / Meg3, H19, linc-MD1, lncRNA containing sine, Yam1, lncRNA-YY1, LncMyoD, MALAT1, Dum, MUNC, Linc-RAM and Lnc-mg, lncRNA SYISL. These lncRNAs regulate muscle development and regeneration through various mechanisms [13–19]. For example, skeletal muscle-specific overexpression of lnc-mg promotes muscle hypertrophy and increases muscle mass [18]. linc-MD1 plays a role in the differentiation time of human myoblasts and its level in Duchenne muscle cells is significantly reduced [20]. As compared to wild type (WT) mice, SYISL-KO mice have more muscle mass and greater muscle fiber density [19]. However, most roles of lncRNAs are identified using microarrays; few studies applied high-throughput sequencing and single-nuclear RNA sequencing (snRNA-seq) analysis in muscles [21, 22]. There is a lncRNA 2310043L19Rik, named lnc-231, has been reported to highly expressed during myoblast differentiation. lnc-231 may regulate skeletal muscle proliferation and differentiation by acting as a ceRNAs [23], but the underlying action mechanism of lnc-231 is not known yet.

miR-125 family consists of three homologs miR-125a, miR-125b-1, and miR-125b-2 [24]. miR-125 family has been widely investigated in many carcinomas or other diseases where it functions either as a promoter or as a repressor [25–27]. Previous studies have shown that miR-125b acts as a negative regulator in skeletal myogenesis [28], while miR-125a-5p regulates MAPK14 transcripts level in extraocular muscles. The pattern of miR-125a-5p expression is fundamentally different from that in limb muscles [29].

Mammalian E2F, a family of transcription factors, regulates cell cycle progression through cooperating with other cell cycle regulatory genes [30]. E2F3 has been demonstrated to be essential for cardiac development and function [31]. The loss of E2F3 impaired the proliferation of vascular smooth muscle cells [32], suggesting that E2F3 plays a promoting role during the proliferation of myoblasts [33].

To study the role of lncRNA 2310043L19Rik in myogenesis, we induced differentiation of C2C12 *in vitro* to find differentially and upregulated lncRNA. The results showed that Lnc-231 was highly expressed in skeletal muscle. Lnc-231 inhibited myoblast differentiation and promote its proliferation via reducing the abundance of miR-125a-5p, which in turn restored the translation level of gene E2F3, and thereby promoting the proliferation of C2C12.

## RESULTS

### Expression pattern of LncRNA 2310043L19Rik

**Figure 1:**
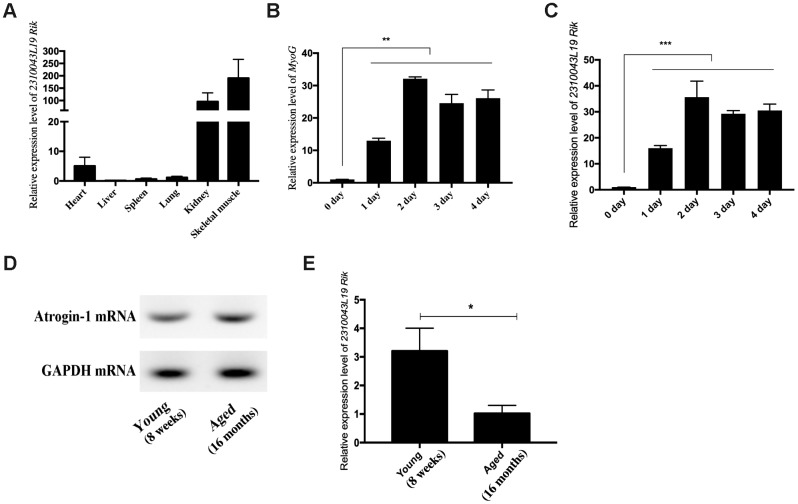
**Expression pattern of lncRNA 2310043L19Rik.** (**A**) Real-time PCR analysis of lncRNA 2310043L19Rik expression in 6 types of mouse tissues. Mean values ± SEM, n=3, *P< 0.05, **P< 0.01. Mice were 8-week old. (**B**) Real-time PCR analysis of MyoG expression in C2C12 during 4 days of differentiation. (**C**) Real-time PCR analysis of lncRNA 2310043L19Rik expression in C2C12 during 4 days of differentiation. Mean ±SEM, n=3. (**D**) Semi-quantitative RT-PCR analysis of mRNA expression of Atrogin-1, the muscle atrophy marker gene marker, in the tibialis anterior muscle of young (8 weeks) and aged (16 months). (**E**) Real-time PCR analysis of lncRNA 2310043L19Rik expression in young mouse (8 weeks) and aged (16 months) mice in the tibialis anterior muscle. Mean values ± SEM, n=3, *P< 0.05, **P< 0.01, ***P< 0.001.

We found the differentially expressed lncRNA 2310043L19Rik (lnc-231) from previous sequencing results [17]. Interestingly, tissue expression profiles showed that lnc-231 was highly expressed in the skeletal muscle (Figure 1A). This reminded us that lnc-231 may involve in the development of skeletal muscle. Inducing myoblast into myotube is an effective model in exploring myogenesis. To explore the role of lnc-231 in myogenesis, C2C12 differentiation was successfully induced *in vitro*, as suggested by a significant elevation of MyoG expression (Figure 1B). Meanwhile, the expression of lnc-231 was significantly increased during differentiation (Figure 1C). In addition, qRT-PCR results showed that Atrogin-1, a marker of muscle atrophy, expressed higher in skeletal muscle of aged mice than that in the adults’ (Figure 1D). In contrast, the expression of lnc-231 in aged skeletal muscle is lower than that in adult mice (Figure 1E). Interestingly, these data suggested that the expression level of Atrogin-1 and lnc-231was negatively correlated during myogenesis.

### LncRNA 2310043L19Rik regulates myoblast differentiation

**Figure 2:**
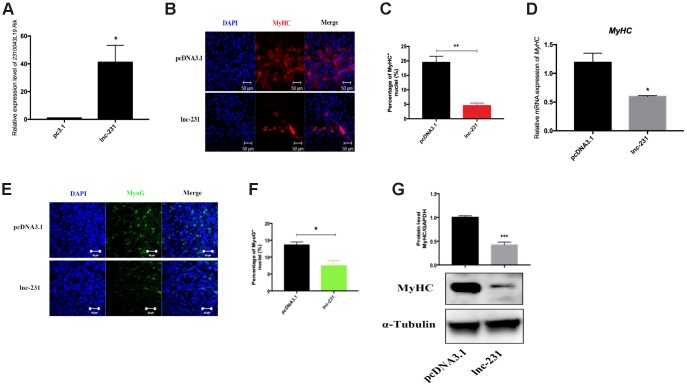
**LncRNA 2310043L19Rik overexpression inhibits myoblast differentiation.** (**A**) Real-time PCR analysis of 2310043L19Rik expression in C2C12 transfected with control pcDNA3.1 or lnc-231. (**B**) Representative photographs of MyHC immunofluorescence staining in C2C12 cells differentiated for 4 d showing that lnc-231 inhibited myoblast differentiation. Positively stained cells were quantified (**C**). (**D**) Real-time PCR analysis of MyHC expression in C2C12 transfected with control pcDNA3.1 or lnc-231 then cultured in DM for 4 days. (**E**) Representative photographs of MyoG immunofluorescence staining in C2C12 cells differentiated for 4 d showing that lnc-231 inhibited myoblast differentiation. Positively stained cells were quantified (**F**). (**G**) Western blot results showing that 2310043L19Rik overexpression significantly decreased the protein expression levels of MyHC in C2C12 cells differentiated for 3 d, and relative protein lever was performed.

**Figure 3:**
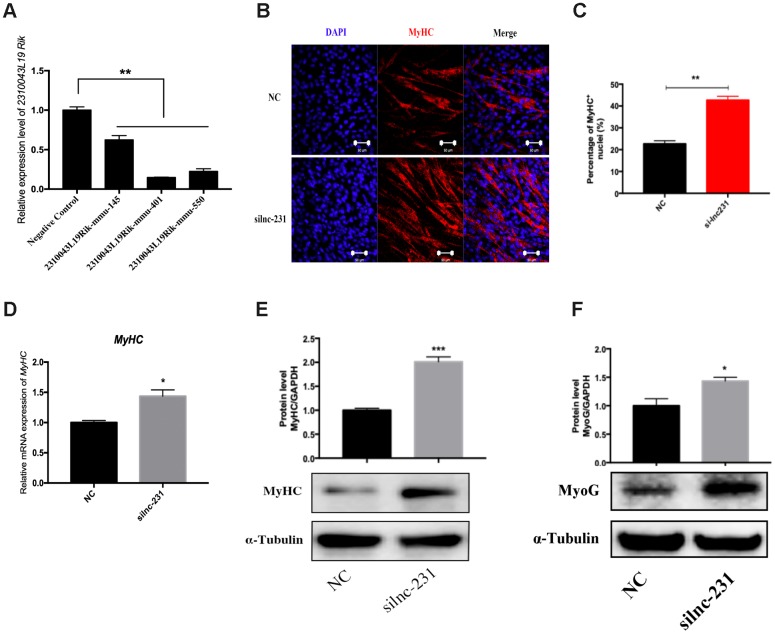
**LncRNA 2310043L19Rik knockdown promotes myoblast differentiation.** (**A**) Real-time PCR analysis of 2310043L19Rik knockdown in C2C12 transfected with control NC or three silnc-231s. (**B**) Representative photographs of MyHC immunofluorescence staining in C2C12 cells differentiated for 4 d transfected with control NC or silnc-231 showing that lnc-231 promoted myoblast differentiation. Positively stained cells were quantified (**C**). (**D**) Real-time PCR analysis of MyHC expression in C2C12 transfected with control NC or silnc-231 then cultured in DM for 4 days. Western blot results showing that 2310043L19Rik knockdown significantly increased the protein expression levels of MyHC (**E**) and MyoG (**F**) in C2C12 cells differentiated for 3 d, and relative protein levels were measured.

To further explore the function of lnc-231 on myoblasts, we constructed an expressing vector encoding lnc 231 (Figure 2A). Overexpression of lnc-231 inhibited the expression of MyHC and MyoG (Figure 2B, 2C, 2E, 2F). In addition, the lnc-231 expressing vector significantly reduced MyHC mRNA (Figure 2D) and protein levels (Figure 2G). Moreover, lnc-231 overexpression inhibited the transition of myoblasts into the multi-nuclear myotubes and suppressed their differentiation. To further investigate the role of lnc-231, we designed three siRNAs against lnc-231. Knock down analysis showed that approximate 80% of lnc-231 was silenced upon siRNAs treatment (Figure 3A), which was followed by an elevation of MyHC positive cell rates (Figure 3B, 3C), MyHC mRNA/protein level, and MyoG protein level (Figure 3D–3F).

### LncRNA 2310043L19Rik regulates G_0_/G_1_ to S transition and proliferation of myoblasts

**Figure 4:**
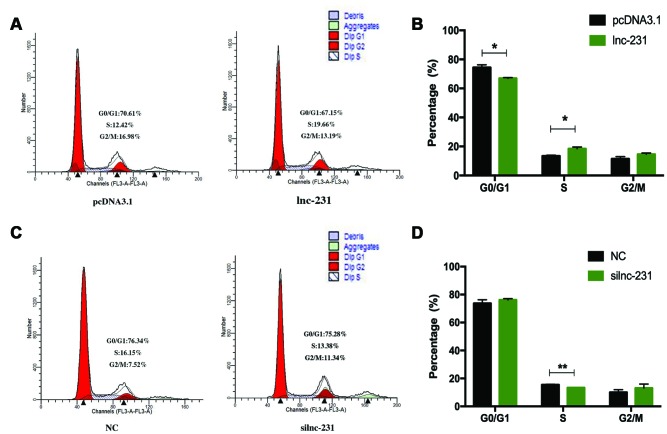
**LncRNA 2310043L19Rik regulates G_0_/G_1_ to S transition.** (**A**) C2C12 cells were transfected with pcDNA3.1 or lnc-231 and the cell cycle phase and proliferation index (**B**) were analyzed by propidium iodide flow cytometry. (**C**) C2C12 cells were transfected with siln-231 or siNC and the cell cycle phase and proliferation index (**D**) were analyzed by propidium iodide flow cytometry.

**Figure 5:**
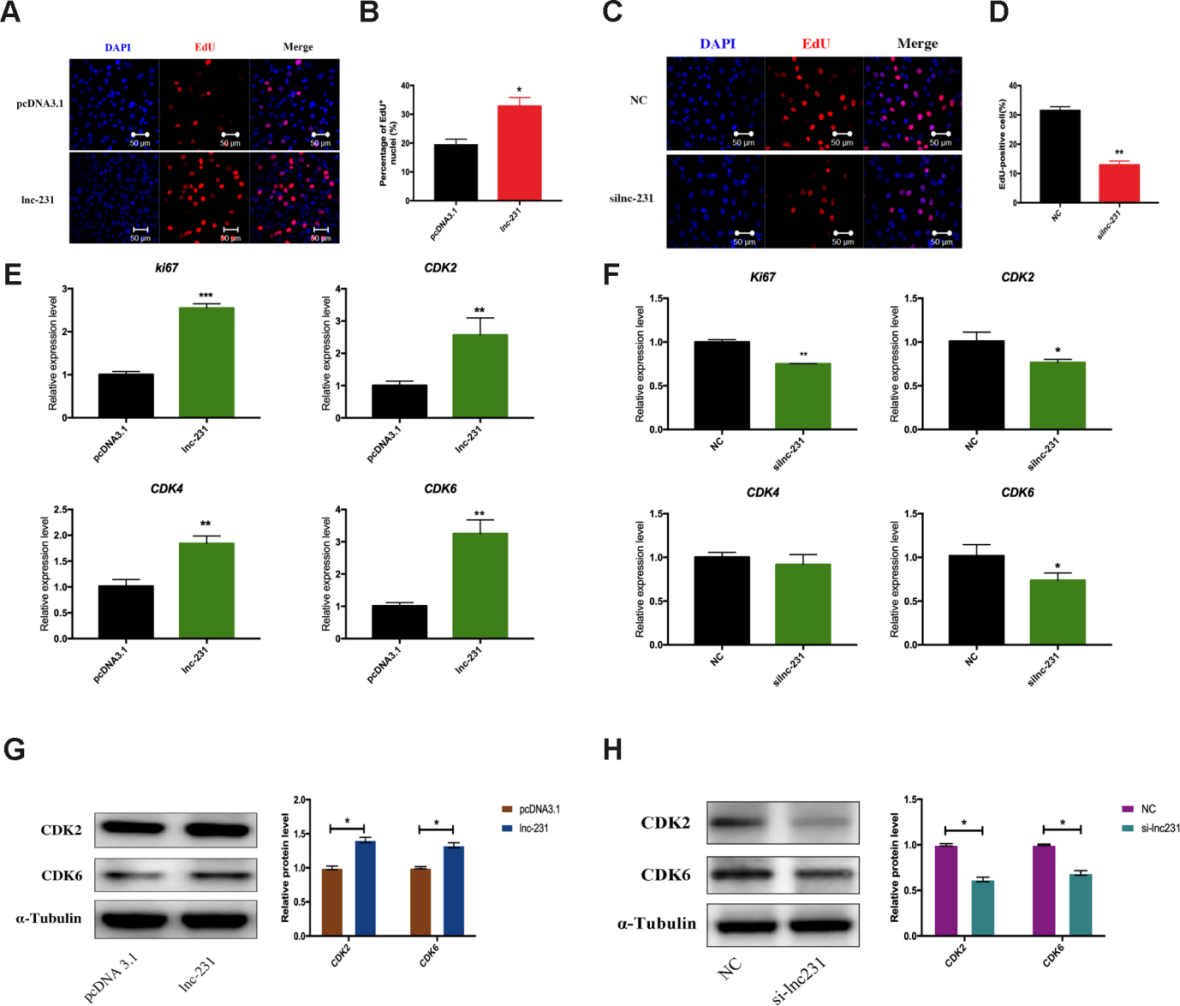
**LncRNA 2310043L19Rik induces G_0_/G_1_ to S transition and cell proliferation.** (**A**) Proliferating C2C12 cells were labeled with EdU after transfection with 2310043L19Rik overexpression vector (lnc-231), control vector (pcDNA3.1) according to the manufacturer’s instructions. The cell nuclei were stained with DAPI. The percentage of EdU^+^ cells was quantified (**B**). (**C**) Proliferating C2C12 cells were labeled with EdU after transfection with 2310043L19Rik inhibitor (silnc-231), or siNC. The cell nuclei were stained with DAPI. The percentage of EdU^+^ cells was quantified (**D**). (**E**) Real-time PCR analysis of Ki67 and cell cycle-dependent kinases (*CDK2, CDK4, CDK6*) mRNA expression in C2C12 transfected with control pcDNA3.1 or lnc-231 then cultured in GM for 24 h. (**F**) Real-time PCR analysis of Ki67 and cell cycle-dependent kinases (*CDK2, CDK4, CDK6*) expression in C2C12 transfected with siNC or silnc-231 cultured in GM for 24 h. Western blot results analysis of 2310043L19Rik overexpression (**G**) and knockdown (**H**) on the protein expression levels of CDK2 and CDK6 in C2C12 cells cultured in GM for 48 h.

In the propidium iodide flow cytometry assays, overexpression of lnc-231 significantly increased the S phase cell ratio (Figure 4A, 4B). On the contrary, the ratio of cells in the S phase decreased by knocking down lnc-231 (Figure 4C, 4D). These data indicated that lnc-231 might play a positive role in regulating the G_0_/G_1_ to S transition of C2C12 cell cycle. Furthermore, overexpression of lnc-231 elevated the rate of EdU positive cells (Figure 5A, 5B), while knockdown of lnc-231 caused a decrease in the rate of EdU-positive cells (Figure 5C, 5D). These results suggested that lnc-231 might stimulate proliferation of myoblast. In addition, we detected the expression of the key proliferation marker gene Ki67 and cyclin-dependent kinases (CDK2, CDK4, and CDK6). qRT-PCR results showed increased mRNA levels of Ki67, CDK2, CDK4, and CDK6 in cells with lnc-231 overexpression (Figure 5E). In contrast, mRNA levels of Ki67, CDK2, CDK6 were reduced after silencing lnc-231 (Figure 5F). Western blot results showed overexpression of lnc-231 raised protein levels of CDK2 and CDK6 (Figure 5G), but suppressed expression of CDK2 and CDK6. In summary, these findings suggested that lnc-231 might promote cell proliferation by inducing cell cycle progression from G_0_/G_1_ to S through upregulating the expression of cell cycle-dependent kinases (CDK2/4/6).

### LncRNA 2310043L19Rik regulates the expression of E2F3 by sponging miR-125a-5p

**Figure 6:**
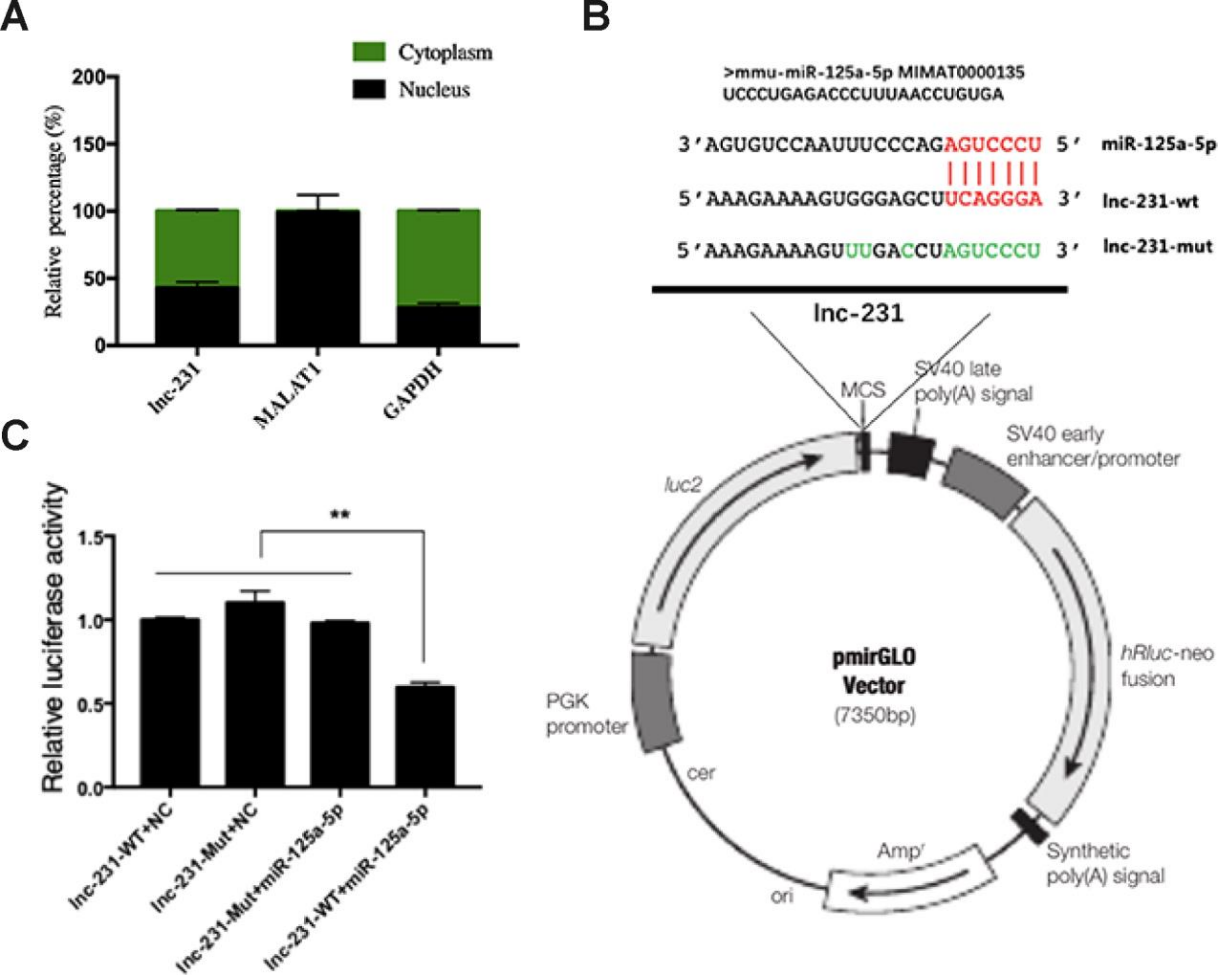
**Identification of functional binding sites of miR-125a-5p in the lncRNA 2310043L19Rik.** (**A**) Result of real-time qPCR showed 2310043L19Rik, MALAT1, GAPDH to be present in both cytoplasm and nucleus of C2C12 cells. (**B**) The mutant sequences of lnc-231-Mut and miR-125a-5p mimics; green: mutant nucleotides. (**C**) miR-125a-5p mimic, NC mimic, and were co-transfected with plasmid pmirGLO-2310043L19Rik luciferase vector (lnc-231-WT) or pmirGLOAK017368-Mut vector (lnc-231-Mut) into C2C12 cells, and the normalized relative luciferase activities (Renilla/firefly) were analyzed.

**Figure 7:**
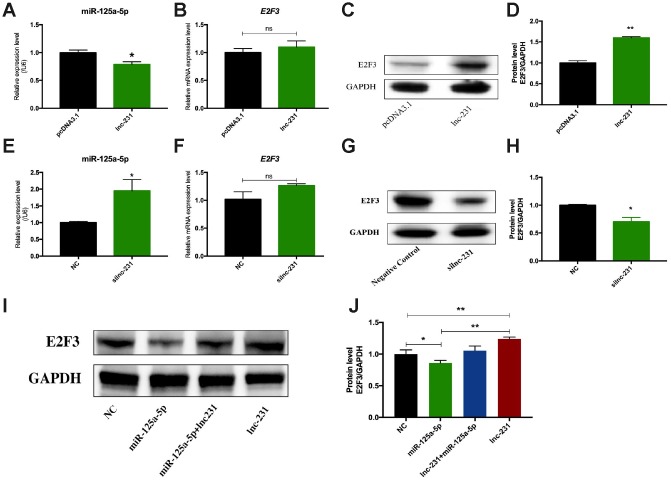
**LncRNA 2310043L19Rik regulates the expression of E2F3 by sponging miR-125a-5p.** Real-time PCR analysis of miR-125a-5p expression in C2C12 transfected with and 2310043L19Rik overexpression (**A**) (pcDNA3.1, lnc-231), and 2310043L19Rik knockdown (**E**) (siNC or silnc-231) cultured in GM for 24 h. Real-time PCR analysis of E2F3 mRNA expression with 2310043L19Rik overexpression (**B**) (in C2C12 transfected with pcDNA3.1, lnc-231), and 2310043L19Rik knockdown (**F**) (in C2C12 transfected with siNC or silnc-231) cultured in GM for 24 h. Western blot results analysis of 2310043L19Rik overexpression (**C**) and knockdown (**G**) affecting on the protein expression levels of E2F3 in C2C12 cells cultured in GM for 48 h. and relative protein lever was performed (**D**, **H**). (**I**) Western blot results analysis of E2F3 in co-transfection assay, and quantification of the E2F3 expression (**J**).

To further explore the regulatory mechanism of lnc-231, we performed a nuclear separation experiment and found that cytoplasmic expression of lnc-231 was higher than that in the nucleus (Figure 6A). This suggests that lnc-231 may act as a ceRNA by sponging miRNAs in the cytoplasm. Using bioinformatics analysis, we predicted miRNAs that bind to lnc-231, and identified miR-125a-5p as a candidate miRNA. To further verify whether lnc-231 is targeted by miR-125a-5p, we constructed a luciferase reporter vector ligating with lnc-231-wt and lnc-231-mut (Figure 6B). The results showed that the luciferase activity was significantly decreased in the lnc-231-WT and miR-125a-5p co-transfected groups (Figure 6C, showing that miR-125a-5p binds to lnc-231-WT. It has been reported that the E2F3 is a target gene of miR-125a-5p, which can inhibit the protein level of E2F3 and inhibit the proliferation of myoblasts [34]. Our results showed that the alterations of miR-125a-5p abundance affected either by overexpressing or knocking down lnc-231 (Figure 7A, Figure 7E) had no effect on the expression of E2F3 mRNA (Figure 7B, 7F). However, overexpression of lnc-231 improved the protein level of E2F3 (Figure 7C, Figure 7D), while lnc-231 silencing suppressed the protein level of E2F3 (Figure 7G, 7H). To assess whether lnc-231 might regulate the protein level of E2F3 via sponging miR-125a-5p, we designed a co-transfection assay. The results showed (Figure 7I, 7J) that overexpression of miR-125a-5p inhibited E2F3 protein level and the decreased E2F3 protein level could be rescued by co-overexpression of lnc-231.

### LncRNA 2310043L19Rik adsorbs miR-125a-5p to promote myoblast proliferation

**Figure 8:**
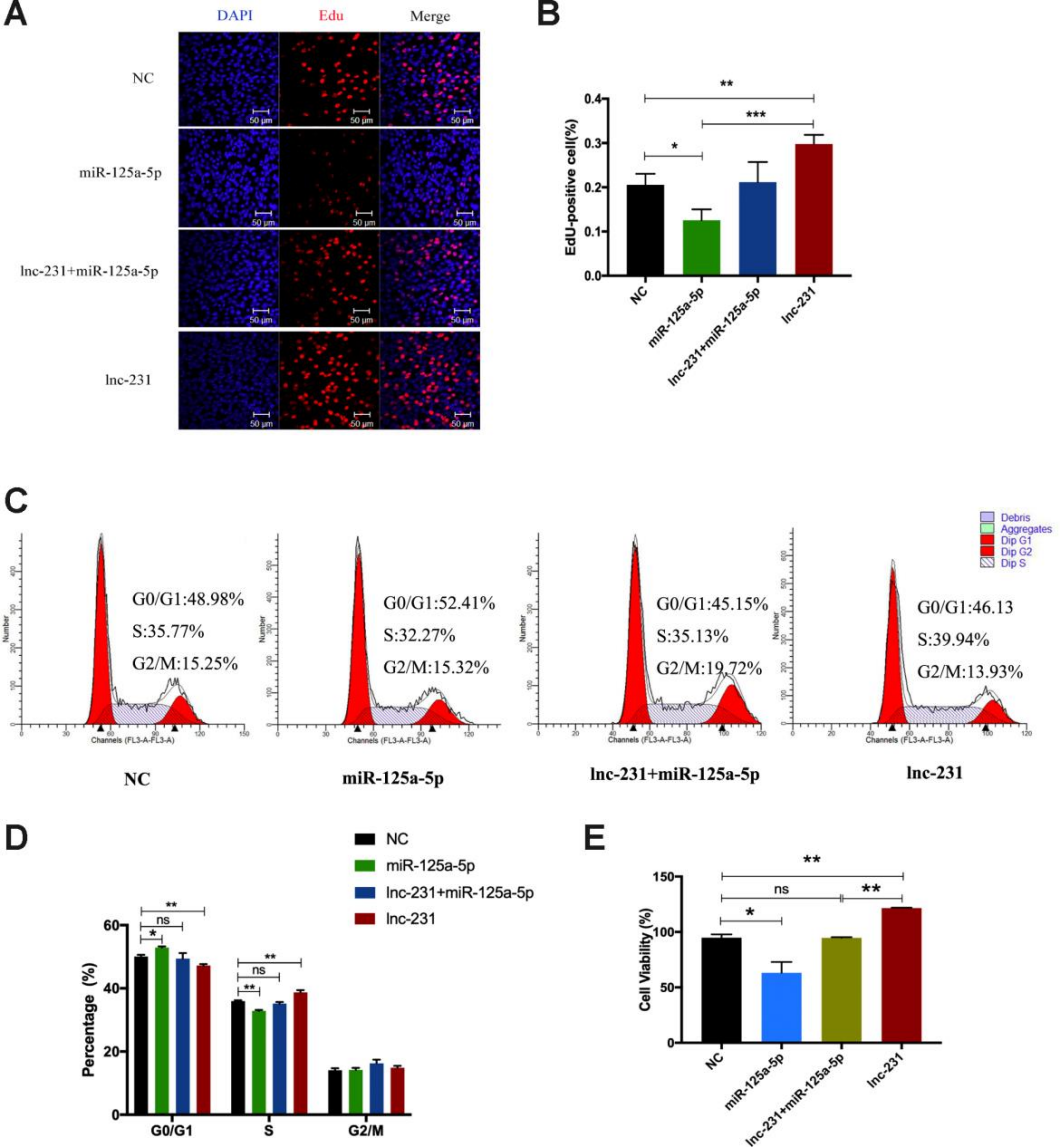
**lncRNA 2310043L19Rik promotes proliferation of myoblast cells by attenuating function of miR-125a-5p.** (**A**) Proliferating C2C12 cells were labeled with EdU after transfection with control NC (pcDNA3.1+ mimics NC), mimics miR-125a-5p, mimics miR-125a-5p+lnc-231, lnc-231. The cell nuclei were stained with DAPI. The percentage of EdU^+^ cells was quantified (**B**). (**C**) Flow cytometry analysis of the percentage of S-phase cells after transfection with control NC (pcDNA3.1+ mimics NC), mimics miR-125a-5p, mimics miR-125a-5p+lnc-231, lnc-231. Statistical analysis of S-phase cells ratio (**D**). (**E**) The determination of cell viability using the CCK-8 assay. Mean values ± SEM, n=3, *P< 0.05, **P< 0.01.

The results of EdU assay showed (Figure 8A) that overexpression of miR-125a-5p significantly reduced the rate of EdU-positive cells (Figure 8B). Compared with miR-125a-5p overexpression alone, co-overexpression of lnc-231 and miR-125a-5p elevated the rate of EdU positive cells. The rate of EdU-positive cells in lnc-231 overexpressed cells was higher than that co-overexpressed with lnc-231 plus miR-125a-5p. Flow cytometry results showed that overexpression of lnc-231 increased the percentage of S phase cells (Figure 8C, 8D). Overexpression of miR-125a-5p arrested cells in the G_0_/G_1_ phase and reduced the population in the S phase, while Co-overexpression of lnc-231 and miR-125a-5p increased the fraction of S phase cells.

## DISCUSSION

**Figure 9:**
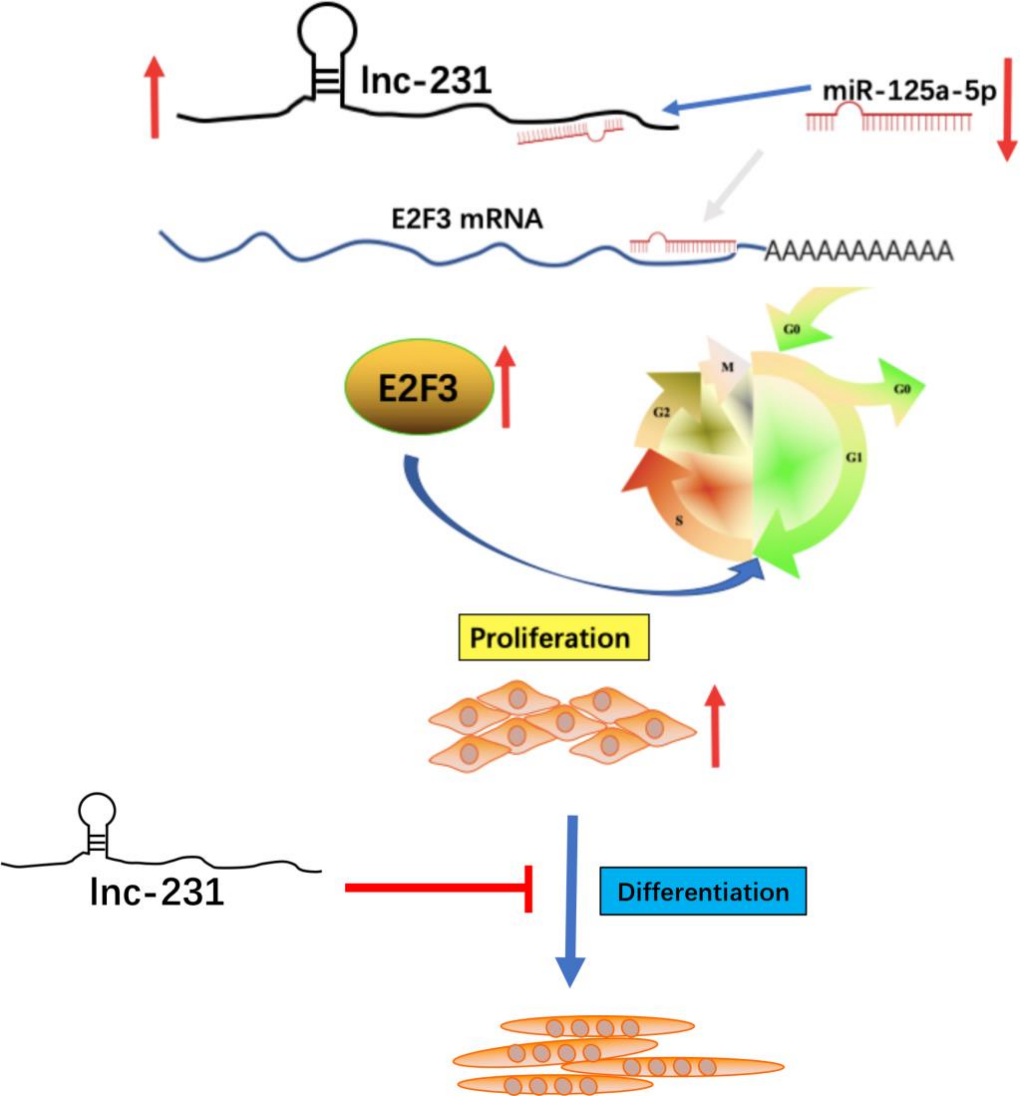
**A schematic model of lncRNA 2310043L19Rik-mediated proliferation of myoblast through sponging miR-125a-5p.** In brief, E2F3 can promote proliferation of C2C12 myoblasts. In addition, miR-125a-5p inhibits E2F3 expression and attenuates the function of E2F3 by directly targeting the 3’-UTR of E2F3. lncRNA 2310043L19Rik inhibits differentiation through a certain pathway. lncRNA 2310043L19Rik promotes proliferation of myoblast cells by attenuating function of miR-125a-5p.

In this study, we demonstrated that a long non-coding RNA 2310043L19Rik(lnc-231) is highly expressed in skeletal muscle, promotes proliferation and inhibits myoblast differentiation. Lnc-231 acts as a ceRNA to sponge miR-125a-5p and negatively regulates the abundance of miR-125a-5p in myoblasts, resulting in the restoration of protein expression of its target gene E2F3, and thereby promoting the cell cycle progression of muscle cells by inducing G_0_/G_1_ to S transition (Figure 9).

It has been reported that lnc-231 is significantly elevated during muscle cell differentiation. [17]. This is consistent with our tissue expression profiling results, which showed that lnc-231 had higher expression in skeletal muscle than in other tissues, suggesting that lnc-231 may be involved in the regulation of skeletal muscle development. Interestingly, the muscular expression of lnc-231 was significantly higher in young mice than that in aged mice; however the expression of Atrogin-1 (a marker gene of muscle atrophy) in aged mice was significantly higher than that in young mice. Since apoptosis of skeletal muscle cells is believed to cause muscle atrophy [35–37], we speculated that lnc-231 may inhibit the apoptosis of skeletal muscle cells, although the specific mechanism remains to be uncovered.

We also found that overexpression of lnc-231 inhibited myoblast differentiation, while knockdown of lnc-231 promoted myoblast differentiation. In agreement with this, lncRNA *SYISL* has been reported to promote myoblast proliferation and fusion but inhibit myogenic differentiation through interacting with polycomb repressive complex 2 [19]. The inhibitory effects of lncRNA on differentiation might be achieved via absorbing several types of differentiation-related miRNAs [38]. Therefore, the specific mechanism of lnc-231-induced inhibition of differentiation still needs further investigations.

In addition, our results showed that lnc-231 promoted the G_0_/G_1_-to-S phase progression in myoblasts, and hence promoting cell proliferation. Using bioinformatics analysis and luciferase reporter vector assays, we demonstrated that lnc-231 can serve as a direct target for miR-125a-5p. As a ceRNA, it can absorb miR-125a-5p to reduce the cytoplasmic abundance of miR-125a-5p, and increase the expression of E2F3 protein. It has been reported that miR-125a-5p targets E2F3 to inhibit the proliferation of myoblasts. Our results showed that miR-125a-5p overexpression inhibited the expression of E2F3 protein levels, which is consistent with previous studies [34].

In conclusion, lnc-231 inhibits myoblast differentiation, and serves as a ceRNA that adsorbs miR-125a-5p to change its enrichment, thereby promoting the translation of the target gene E2F3, which in turn promotes cell cycle progression by activating the G_0_/G_1_ to S transition. Our results suggested the role of lnc-231/miR-125a-5p/E2F3 pathway in regulating myoblast proliferation and differentiation. Indicatively, further investigations of this pathway might contribute to a better understanding of the mechanisms underlying muscle atrophy and skeletal muscle regeneration in the future.

## MATERIALS AND METHODS

### Ethics statement

All experimental procedures on the mice were conducted according to the Guidelines for Experimental Animals of the Ministry of Science and Technology (Beijing, China). The study protocol was reviewed and approved by the ethics committee of Nanjing Agricultural University. Animals were humanely euthanized as necessary, to reduce suffering.

### Bioinformatics analyses

The sequence of lncRNA 2310043L19Rik was taken from the National Center for Biotechnology Information Search database (Bethesda MD, USA) genome browser (https://www.ncbi.nlm.nih.gov/pubmed/). lncRNA 2310043L19Rik (genomic size: 699 bp), GenBank: AK009784.1 of the mouse genome. Putative target sites of 2310043L19Rik are predicted on miR and a website (http://www.microrna.org/microrna/home.do/; Memorial Sloan Kettering Cancer Center, New York, NY, USA). The target genes of miR-125a-5p were predicted by TargetScan (http://www.targetscan.org/vert_71/). E2F3 has been identified as a target gene of the miR-125a-5p [34], so and we focused on E2F3 in this study.

### Animals and cells

BALB/c mice were obtained from Qinglongshan Laboratory Animal Company (Nanjing, China). Mice were housed in a room of Nanjing Agricultural University under conventional conditions with appropriate temperature and humidity and fed a standard diet. Four mice at 8 weeks of age were used to collect different tissues to measure the expression of 2310043L19Rik by quantitative PCR (qPCR). Mouse C2C12 cells were cultured in growth medium (GM) (26) consisting of 89% high-glucose DMEM, 10% (v/v) fetal bovine serum, and 1% penicillin-streptomycin (both from Thermo Fisher Scientific) at a constant temperature of 37°C in 5% CO_2_. Similar to induction of differentiation of C2C12 cells, mouse C2C12 myoblasts were induced in differentiation medium (DM) consisting of 97% DMEM, 2% horse serum (Thermo Fisher Scientific), and 1% penicillin–streptomycin.

### Overexpression constructs, siRNA, and transfections

Constructs overexpressing 2310043L19Rik (lnc-231) were obtained by cloning 2310043L19Rik cDNA into a pcDNA3.1^+^ plasmid (Thermo Fisher Scientific). siRNA molecules against 2310043L19Rik (silnc-231), NC silnc-231, a miR-125a-5p mimic, a miR-125a-5p NC mimic (NC mimic), were designed and synthesized by GenePharma (Shanghai, China). Mouse C2C12 myoblasts were transfected with plasmid DNA with Lipofectamine™ 3000 Transfection Reagent (Invitrogen, USA). All transfections were performed according to the manufacturer’s specifications.

To determine the effects of 2310043L19Rik on proliferation and differentiation of C2C12 cells, C2C12 myoblasts were transfected with a 2310043L19Rik overexpression vector (lnc-231), control vector (pcDNA3.1), siRNA molecules against silnc-231, NC silnc-231. To study the influence of 2310043L19Rik on miR-30c and E2F3 we transfected lnc-231, silnc-231, miR-125a-5p mimic, silnc-231, and their NCs into C2C12 cells as proliferating myoblasts in GM for 48 h or differentiated myoblasts (after 12 h of transfection, cells were induced in DM for 72 h) in several groups: the lnc-231 overexpression group transfected with pcDNA3.1, lnc-231, respectively; the silnc-231 group, transfected with NC silnc-231, silnc-231, respectively; the co-transfection group, transfected with pcDNA3.1+ NC mimic, miR-125a-5p, lnc-231+ miR-125a-5p, and lnc-231, respectively. Each group was transfected with equal amounts of plasmid, synthetic RNA, and Lipofectamine™ 3000, to abolish the differences caused by the transfection process.

### qPCR

Total RNA was collected from mouse tissues and C2C12 cells by using Trizol reagent (Thermo Fisher Scientific). Next, the concentration and integrity of RNA were assessed with NanoDrop 2000 (Thermo Fisher Scientific) and denatured gel electrophoresis, respectively. cDNA synthesis for mRNA and miRNA detection was performed with PrimeScript Real Time Master Mix (Perfect Real Time; Takara Biotech, Dalian, China) and Mix-X miRNA First-Strand Synthesis Kit (TaKaRa, Dalian, China), respectively. qPCR for mRNA and miRNA detection was qRT-PCR was performed using AceQ qPCR SYBR Green Master Mix (Vazyme, Nanjing, China) for quantitative on a Step-One Plus Real-Time PCR System (Applied Biosystems, Carlsbad, CA, USA). The primers for quantitative analysis were shown in Table 1. The relative expression levels were calculated using the 2^ΔΔct^ method, and mouse *GAPDH* and *U6* snRNA was used for normalization of mRNA and miRNAs expression levels as endogenous reference genes. Statistical analysis was performed using Prism 6 software (GraphPad Software, San Diego, CA), and data were expressed as mean ± SE unless otherwise noted. Differences were tested using two-tailed Student’s t test and ANOVA test. *P* < 0.05 was considered significant difference.

**Table 1:** Primers used for qRT-PCR analysis.

**Gene name**	**Forward primer**	**Reverse primer**
2310043L19Rik	GCACCTTGAGTGGTGACAGGGC	ACACAGAGGTGGGCCTCAACGT
MyoG	GAGACATCCCCCTATTTCTACCA	GCTCAGTCCGCTCATAGCC
Ki67	CGCAGGAAGACTCGCAGTTT	CTGAATCTGCTAATGTCGCCAA
CDK2	GCGACCTCCTCCCAATATCG	GTCTGATCTCTTTCCCCAACTCT
CDK4	CTGGAAGAAGTCTGCGTCGG	GTCTTGCCAAAGCGGTTCAG
CDK6	TCTCACAGAGTAGTGCATCGT	CGAGGTAAGGGCCATCTGAAAA
GAPDH	ATCACTGCCACCCAGAAGACT	CATGCCAGTGAGCTTCCCGTT
E2F3	CAGATCCTCACTACGAACCCT	GTTCCAGCCTTCGCTTTGC
Atrgin-1	CAGCTTCGTGAGCGACCTC	GGCAGTCGAGAAGTCCAGTC
MyHC	GGCAGTCGAGAAGTCCAGTC	TGTCGTACTTGGGCGGGTTC
miR-125a-5p	ACTACATCCCTGAGACCCTTTAAC	CAGTGCGTGTCGTGGAGT
U6	CTCGCTTCGGCAGCACA	AACGCTTCACGAATTTGCGT

### Cytoplasmic and nuclear lncRNA *2310043L19Rik*

RNAs were extracted from C2C12 cells by using a Nuclear or Cytoplasmic RNA Purification Kit (Thermo Fisher Scientific), according to the manufacturer’s instructions. First, the cell pellet was resuspended in buffers from an RNA Purification Kit and centrifuged twice at 4°C. The supernatant was taken as the cytoplasmic fraction, and the pellet was saved as the nuclear fraction. RNAs of cytoplasmic and nuclear fractions were extracted with Trizol reagent. The relative levels of lncRNA 2310043L19Rik in both cytoplasmic and nuclear fractions were analyzed by Realtime qPCR.

### 5-Ethynyl-2′-deoxyuridine assay

Near-confluent C2C12 cells (80–90%) were transfected with a vector overexpressing 2310043L19Rik (lnc-231), a control vector (pcDNA3.1), silnc-231, or NC silnc-231 (double strand). And the co-transfection group, transfected with pcDNA3.1+ NC mimic, miR-125a-5p, lnc-231+ miR-125a-5p, and lnc-231, respectively. After 12 h of transfection, C2C12 cells were cultured in fresh GM that contained 5-ethynyl-29-deoxyuridine (EdU; final concentration, 10 mM) for 4 h. An EdU assay with the Cell-Light EdU Apollo567 In Vitro Kit 100T (RIBOBIO, Guangzhou, China) was performed to test the proliferation of C2C12 cells and analyzed by confocal microscopy (LSM 700 META; Zeiss, Jena, Germany) to detect the EdU stained cells.

### Cell cycle flow cytometry

Cell cycle and cell viability analyses were performed in accordance with the manufacturer’s instructions. were transfected with a vector overexpressing 2310043L19Rik(lnc-231), a control vector (pcDNA3.1), silnc-231, or NC silnc-231 (double strand). And the co-transfection group, transfected with pcDNA3.1+ NC mimic, miR-125a-5p, lnc-231+ miR-125a-5p, and lnc-231, respectively. After 36 h of transfection, C2C12 cells were fixed in 70% (v/v) ethanol at -20°C for 12 h. The cells were incubated in 50 mg/ml propidium iodide solution [100 mg/ml RNase A and 0.2% (v/v) Triton X-100] in the dark for 30 min at 4°C. The cells were sorted with a FACSCalibur flow cytometer (BD, Franklin Lakes, NJ, USA), and the data were analyzed by ModFit software (Verity Software House, Topsham, ME, USA). The proliferation index stood for the proportion of mitotic cells in a total of 10,000-20,000 cells [39]. The index was calculated as cell population (100%) (S+G_2_)/(G_0_/G_1_+S+G_2_), with S and G indicating the growth and synthesis phases of the cell cycle.

### Dual-luciferase reporter assays

C2C12 cells were cultured (8×10^4^ cells/cm^2^) in 12-well plates. When the cells reached 70–80% confluence, 50 nM of the pmirGLO-lnc-231 luciferase vector (lnc-231-WT) or pmirGLO-lnc-231 mutant vector (lnc231-Mut), as well as 50 nM of NC, miR-125a-5p mimic were transfected by using Lipofectamine™ 3000 Transfection Reagent. After 24 h of transfection, the cells were harvested, and luciferase assays were performed with the Dual-Luciferase Reporter Assay System (Promega, Madison, WI, USA). For each sample, we normalized Renilla luciferase activity to firefly luciferase expression to account for differences in transfection efficiency.

### Western blot analysis

The cellular protein lysis in ice-cold RIPA (Beyotime, Shanghai, China) buffer containing 1% PMSF (Beyotime, Shanghai, China) was collected after high-speed centrifugation (12,000×g) for 10 min at 4°C. The protein concentration was detected with a BCA Protein Assay Kit (Beyotime, Shanghai, China). The protein extracts were added with 5×SDS-PAGE Sample Loading Buffer (Beyotime, Shanghai, China) and then denatured on a PCR instrument at 100°C for 10 min. The primary antibodies used are as follows: myosin heavy chain (MyHC; dilution 1:200; Developmental Studies Hybridoma Bank, Iowa, USA) and MyoG antibody (1:500, ABclone, Wuhan, China); CDK2, CDK6, E2F3(dilution 1:500; ABclone, Wuhan, China); α-Tubulin and GAPDH (dilution 1:2000; Cell Signaling Technology, Danvers, MA, USA). Horseradish peroxidase–labeled anti-rabbit/goat IgG secondary antibody (dilution 1:5000; Cell Signaling Technology, USA) was used to detect protein expression. Proteins were separated by SDS-PAGE [40], followed by transfer to PVDF membranes (Millipore-Sigma, Billerica, MA, USA), for immunoblot assay with antibodies. Images of targeted proteins were acquired on a chemiluminescence detection system (GE Healthcare, Piscataway, NJ, USA) and analyzed by ImageJ software (National Institutes of Health, Bethesda, MD, USA).

### Immunofluorescence

After the transfection and differentiation processes, C2C12 cells cultured in a 12-well plate in DM for 4 d. After that, the cells were washed with PBS 3 times and fixed in 4% paraformaldehyde for 30 min, followed by 3 washes with PBS. The cells were subsequently incubated in ice-cold 0.5% Triton X-100 at room temperature for 15 min and further washed 3 times. Next, the cells were incubated in blocking solution (1% bovine serum albumin) at room temperature for 2 h. After 3 washes with PBS, the cells were incubated in MyHC and antibody (dilution 1:50; Developmental Studies Hybridoma Bank) and MyoG antibody (1:100, ABclone, Wuhan, China) at 4°C for 12 h. The cells were washed 3 times and incubated with Alexa Fluor 488-Conjugated Goat anti-mouse IgG (H+L) antibody (dilution 1:100; ZSGB-BIO, Beijing, China) and Rhodamine(TRITC)-Conjugated Goat anti-mouse IgG (H+L) antibody (dilution 1:100; ZSGB-BIO, Beijing, China) in the dark. After 1 h of incubation at room temperature, the cells were washed 3 times. The cell nuclei were stained with DAPI in the dark. After 3 washes, images were captured using confocal microscopy (LSM700META; Zeiss).

### Cell viability analysis

Cell viability was determined by WST-8 assay using Cell Counting Kit-8 (CCK-8; Dojindo, CK04). This colorimetric strategy is based on the reduction of tetrazolium salt (WST-8) to a colored formazan product by active dehydrogenases in viable cells. The amount of formazan is therefore directly proportional to the percentage of living cells. Our experimental procedures were performed according to the manufacturer’s instructions. Briefly, C2C12 seeded in 24-well plates were grown to 90% confluency following 2 d of culture, and exposed to the indicated treatments as mentioned above. To measure cell viability, 50 ul of CCK-8 assay solution was added to each well containing 500 ul medium, and incubated at 37°C in a humid atmosphere containing 5% CO^2^ for 2 h. Living cells promoted the formation of formazan, which was detectable at 450 nm under a microplate reader (Bio-Rad, Hercules, CA, USA).

### Statistical analysis

Statistical analysis was performed using SPSS software (Chicago, IL, USA), and graphs were drawn with Prism v.6 (GraphPad Software, La Jolla, CA, USA). Student’s t test was used for group comparisons. Multiple comparisons were performed by one-way ANOVA followed by Dunnett’s *post hoc test*. Results are shown as means±SEM. Statistical significance was set at *P* < 0.05.
